# The Peripheral Role of CCL2 in the Anti-Nociceptive Effect of Sigma-1 Receptor Antagonist BD1047 on Inflammatory Hyperalgesia in Rats

**DOI:** 10.3390/ijms222111730

**Published:** 2021-10-29

**Authors:** Sungkun Chun, Jun-Ho Lee, Seo-Yeon Yoon, Young-Bae Kwon

**Affiliations:** 1 Department of Physiology, Institute for Medical Science, Jeonbuk National University Medical School, Jeonju 54907, Korea; sungkun.chun@jbnu.ac.kr; 2 Department of Anesthesiology and Pain Medicine, Jeonbuk National University Medical School and Hospital, Jeonju 54907, Korea; gojuno@jbnu.ac.kr; 3 Department of Pet Animal, Division of Health and Life Science, Daejeon Institute of Science and Technology, Daejeon 35408, Korea; osoon@hanmail.net; 4 Department of Pharmacology, Institute for Medical Science, Jeonbuk National University Medical School, Jeonju 54907, Korea

**Keywords:** CCL2, Complete Freund’s adjuvant, hyperalgesia, microglia, sigma-1 receptor

## Abstract

Our recent study demonstrated that the CC-chemokine ligand 2 (CCL2) present in primary afferent fibers (PAFs) plays an important role in the microglia-dependent neuronal activation associated with zymosan-induced inflammatory pain. The present study was aimed to evaluate whether BD1047 (a prototypical sigma-1 receptor (Sig-1R) antagonist) is capable of modifying elevated levels of inflammation-evoked CCL2 as a peripheral antinociceptive mechanism. In DRG primary culture, zymosan dose-dependently increased CCL2 release from isolectin B4 (IB4)-positive DRG neurons, a process that was inhibited by co-culture with BD1047. Single treatment of BD1047 before intraplantar injection of zymosan in rats significantly reduced thermal hyperalgesia and mechanical hyperalgesia, as well as CCL2 expression in DRG neurons and microglia activation in the spinal dorsal horn. In the Complete Freund’s adjuvant (CFA)-induced inflammation model, repeated administration of BD1047 dramatically attenuated thermal hyperalgesia and mechanical hyperalgesia, and significantly diminished CCL2 immunoreactivity and microglia activation. Notably, CFA-induced inflammation significantly increased Sig-1R immunoreactivity in DRG neurons, which was co-localized with CCL2 and IB4, respectively. Taken together, our results suggest that BD1047′s anti-nociceptive property was substantially mediated by the inhibition of CCL2 release in unmyelinated PAFs and that this may, in turn, have attenuated the spinal microglia activation that is associated with inflammatory pain.

## 1. Introduction

The sigma-1 receptor (Sig-1R) is associated with a number of neurological disorders that result from dysfunction of the intracellular calcium signaling pathways in ion channels, receptors, or kinases [1]. Intrathecal injection of Sig-1R agonist evokes pain symptoms via calcium-dependent protein kinase and phosphorylation of NMDA receptor NR1 subunit (pNR1) in the spinal cord [2]. A series of experiments confirm that BD1047 (a prototypical Sig-1R antagonist) produces anti-nociceptive effects by modifying neural sensitization (specifically by elevating Fos and pNR1 expression) in response to pain generated by formalin, capsaicin, or dorsal root ganglion (DRG) compression [3,4,5]. While research on the anti-nociceptive mechanisms of Sig-1R antagonists has to date focused on the role of spinal dorsal horn neurons, Sig-1R immunoreactivity is highly expressed in DRG neurons, suggesting that it plays an important peripheral pain perception [6]. Our previous study revealed that Sig-1R immunoreactivity in the spinal cord occurs predominantly in the central endings of isolectin B4 (IB4)-positive unmyelinated primary afferent fibers (PAFs) as well as in spinal neurons (NeuN-positive), but not in microglia or astrocytes after zymosan-induced inflammation [7]. Notably, intraplantar injection of the Sig-1R agonist reverses its anti-nociceptive effect of Sig-1R antagonist against carrageenan-induced inflammatory hyperalgesia [8]. Supporting this, carrageenan-induced peripheral inflammation significantly increases Sig-1R immunoreactivity in IB4-positive DRG neurons [9]. Based on these findings, we assume that peripheral Sig-1R plays an important role in mediating the anti-nociceptive effect of BD1047 by directly regulating peripheral pain triggers in inflammatory conditions.

It is well-established that the immune system, by releasing cytokines, chemokines, and other inflammatory mediators, plays an important role in the development and maintenance of inflammatory pain symptoms associated with rheumatoid arthritis [10]. Among these, chemokines were identified as a possible versatile messenger in neuron–microglia interactions [11]. The intrathecal injection of CC-chemokine ligand 2 (CCL2) into naive rats evoked hyperalgesia via activation of its cognate receptor (CC chemokine family receptor 2 (CCR2) in the spinal cord [12]. We recently also uncovered that zymosan-induced acute inflammation increases the amount of CCL2 released from unmyelinated PAFs, which in turn activates microglia-dependent neuronal activation and hyperalgesia [13]. Complete Freund’s adjuvant (CFA)-induced inflammation also increases CCL2 expression in IB4-positive DRG neurons but not in the spinal cord [14]. Moreover, over-expression of CCL2 amplifies CFA-induced inflammatory pain and related pro-inflammatory cytokine expression [15]. These findings imply that the peripheral elevation of CCL2 plays an important role in the spinal microglia–neuronal interactions associated with both acute and chronic inflammatory pain.

Through our previous studies, we determined that oral administration of BD1047 reduced zymosan-induced acute inflammatory hyperalgesia by inhibiting neuronal activation (via extra-cellular signal-regulated protein kinase) and microglial activation (via phosphorylated p38 mitogen-activated protein kinases (p-p38)) in the spinal cord [7,16]. Until now, the role of CCL2 in BD1047′s anti-nociceptive effect in zymosan-induced hyperalgesia has been unclear. It was suggestive, however, CFA-induced chronic inflammation simultaneously increased both Sig-1R and CCL2 expressions in IB4-positive PAFs [9,14]. Moreover, microglial activation and the production of inflammatory mediators (including CCL2 and interleukin-1β) in the spinal cord both played a critical role in the development of CFA-induced arthritic pain [17]. For these reasons, we hypothesized that the release of CCL2 from inflamed PAFs may be regulated in part by BD1047.

The present study aims to clarify the role of peripheral anti-nociceptive mechanism BD1047, focusing on CCL2 in inflammatory conditions. We first evaluated whether zymosan evokes CCL2 release in DRG primary culture, a release that is otherwise inhibited by co-culture with BD1047 excluding the possibility of a spinal effect of Sig-1R. We then confirmed whether BD1047 alleviates zymosan-induced CCL2 and microglia activation using an in vivo model. We further observed modification of CCL2 elevation and subsequent microglia activation by repeated treatments with BD1047 in a CFA-induced chronic arthritic pain model.

## 2. Results

### 2.1. The Inhibitory Effect of BD1047 on Zymosan-Induced CCL2 Release in DRG Primary Culture

**Figure 1:**
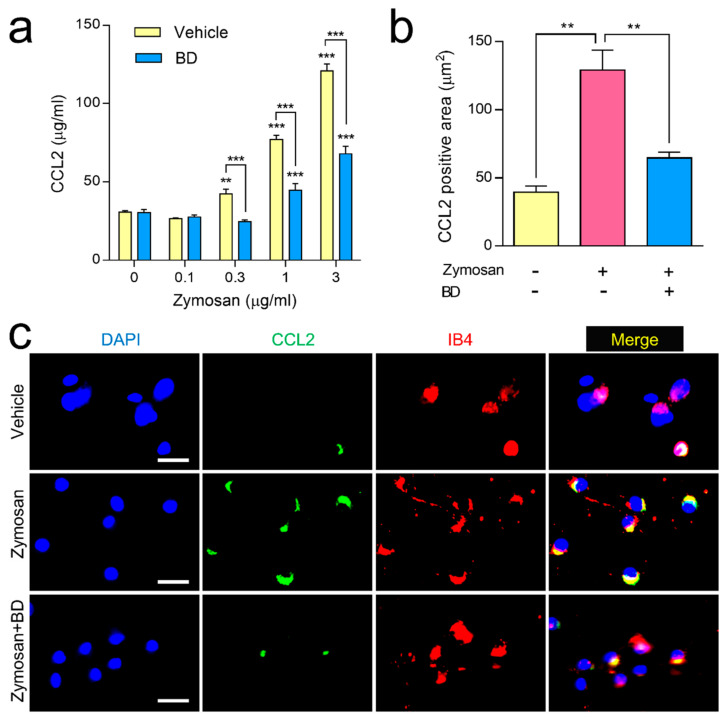
The effect of BD1047 (BD) on zymosan-induced CCL2 release in dorsal root ganglion (DRG) primary culture. (**a**) Zymosan incubation dose-dependently increased CCL2 release in the DRG primary culture cells as compared with vehicle control, which was reversed by BD (10 µM). ** *p* < 0.01, *** *p* < 0.001 (compared with 0 µg/mL of zymosan of each group; data represent the mean ± SEM of three independent experiments; Bonferroni’s multiple comparison test). (**b**) Increased CCL2 immunoreactivity in DRG neurons during zymosan incubation (3 µg/mL) was reversed by BD (10 µM). ** *p* < 0.01 (*n* = 3; data represent the mean ± SEM; Tukey’s multiple comparison test) (**c**) In the triple-labeling study, the zymosan-induced CCL2 immunoreactivity predominantly overlapped with IB4. Scale bar = 50 µm.

In a DRG primary culture study, zymosan (0.1, 0.3, 1 and 3 µg/mL) incubation for 3 h dose-dependently increased the amount of CCL2 released into the culture medium as compared with vehicle control. This release had been significantly inhibited by co-incubation with BD1047 (10 µM) (Figure 1a). Our immunocytochemical experiments revealed that zymosan (3 µg/mL) dramatically elevated CCL2 immunoreactivity in DRG neurons, which had been significantly reduced by co-incubation with BD1047 (10 µM) (Figure 1b,c). A subsequent triple-labeling study revealed that zymosan-induced CCL2 immunoreactivity was predominantly co-localized with IB4-positive DRG neurons (Figure 1c).

### 2.2. The Inhibitory Effect of BD1047 in Zymosan-Induced Acute Inflammatory Pain

**Figure 2:**
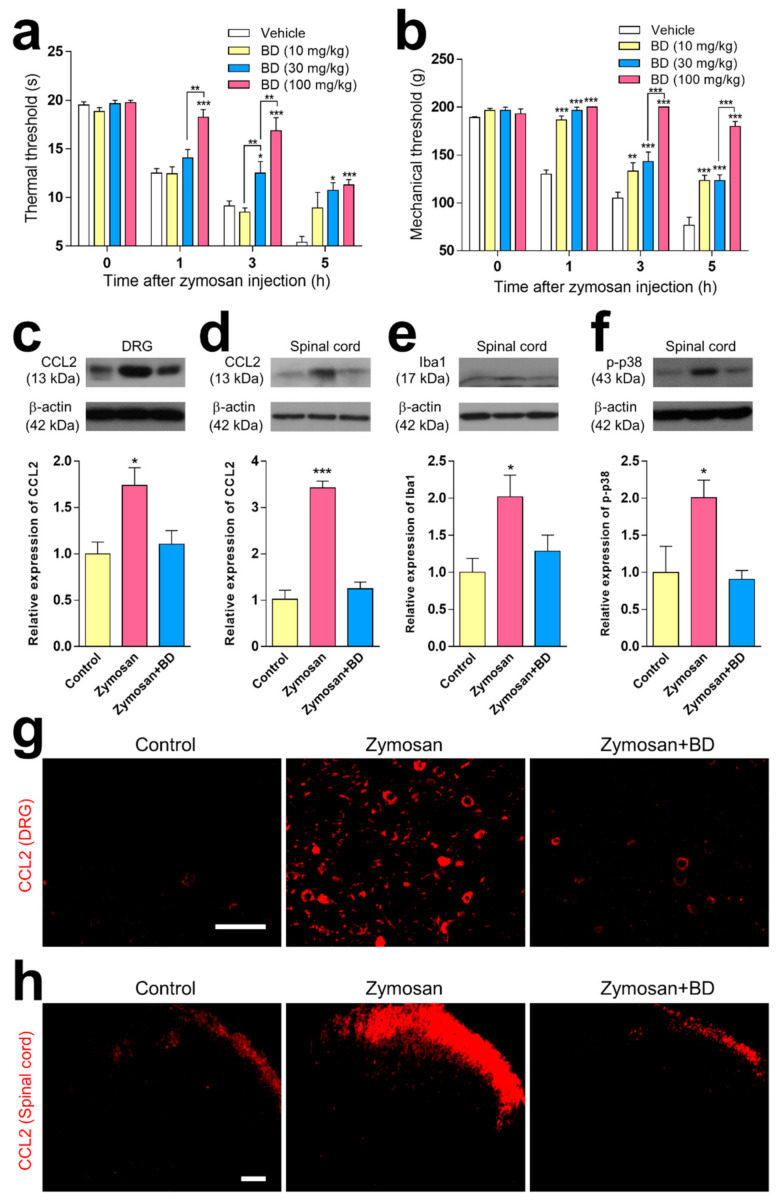
The effect of BD1047 (BD) on zymosan-induced hyperalgesia, and changes in CCL2 elevation and microglial activation. (**a**,**b**) Oral pre-treatment of BD at 1 h before zymosan injection dose-dependently reduces thermal hyperalgesia (**a**) and mechanical hyperalgesia (**b**) as compared with vehicle group. * *p* < 0.05, ** *p* < 0.01, *** *p* < 0.001 (compared with vehicle control group at each time-point; *n* = six rats in each group; data represent the mean ± SEM; Bonferroni’s multiple comparison test). The inhibitory effect of BD (100 mg/kg) on zymosan-induced CCL2 immunoreactivity in both dorsal root ganglion (DRG) (**c**); Western blotting using four rats, (**g**); immunohistochemistry using three rats) and spinal dorsal horn (**d**); Western blotting using four rats, (**h**); immunohistochemistry using three rats). The inhibitory effect of BD on zymosan-induced microglia proliferation (**e**) and phosphorylated p38 mitogen-activated protein kinase (p-p38) expression in spinal dorsal horn (**f**) compared with a non-inflamed control group. Scale bar = 100 µm. * *p* < 0.05, *** *p* < 0.001 (compared from non-inflamed control group; *n* = three rats in each group; data represent the mean ± SEM; Tukey’s multiple comparison test).

A comparison of vehicle groups at the 1, 3 and 5 h after zymosan injection revealed that the oral administration of BD1047 (10, 30, and 100 mg/kg) dose-dependently reduced thermal hyperalgesia (Figure 2a) and mechanical hyperalgesia (Figure 2b). Western blot and immunohistochemical experiments established that CCL2 immunoreactivity was significantly higher in both DRG neurons (Figure 2c,g) and superficial lamina of the spinal cord (Figure 2d,h) 3 h after zymosan injection compared to the non-inflamed control group, and that oral administration of BD1047 (100 mg/kg) significantly reduced this difference. The Western blotting analysis showed that zymosan-induced microglia activation (Iba1, Figure 2e) and p-p38 elevation (Figure 2f) were significantly reduced by the administration of BD1047 (100 mg/kg).

### 2.3. The Effect of Repeated Administration of BD1047 on CFA-Induced Inflammatory Pain

**Figure 3:**
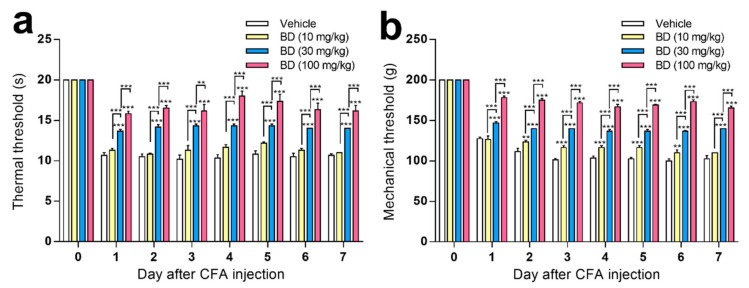
The effect of BD1047 (BD) on complete Freund’s adjuvant (CFA)-induced hyperalgesia. Repeated administration of BD1047 (7 days, daily) dose-dependently inhibited the development of CFA-induced thermal hyperalgesia (**a**) and mechanical hyperalgesia (**b**). ** *p* < 0.01, *** *p* < 0.001 (compared with Vehicle group of each time-point; *n* = six rats in each group; data represent the mean ± SEM; Bonferroni’s multiple comparison test; dose-response analysis using Tukey’s multiple comparison test).

Intra-articular injection of CFA into the right ankle joint produced evident thermal hyperalgesia (Figure 3a) and mechanical hyperalgesia (Figure 3b) in the ipsilateral hind paw, which was apparent from 1 day after CFA-injection. These symptoms were sustained over 7 days, during which time the contralateral hind paw showed no signs of pain (data not shown). Repeated oral treatment of BD1047 (10, 30, and 100 mg/kg) over the 7 days following CFA injection dose-dependently reduced thermal hyperalgesia (Figure 3a) and mechanical hyperalgesia (Figure 3b) in the ipsilateral hind paw.

### 2.4. The Effect of BD1047 on CFA-Induced CCL2 Elevation and Microglial Activation

**Figure 4:**
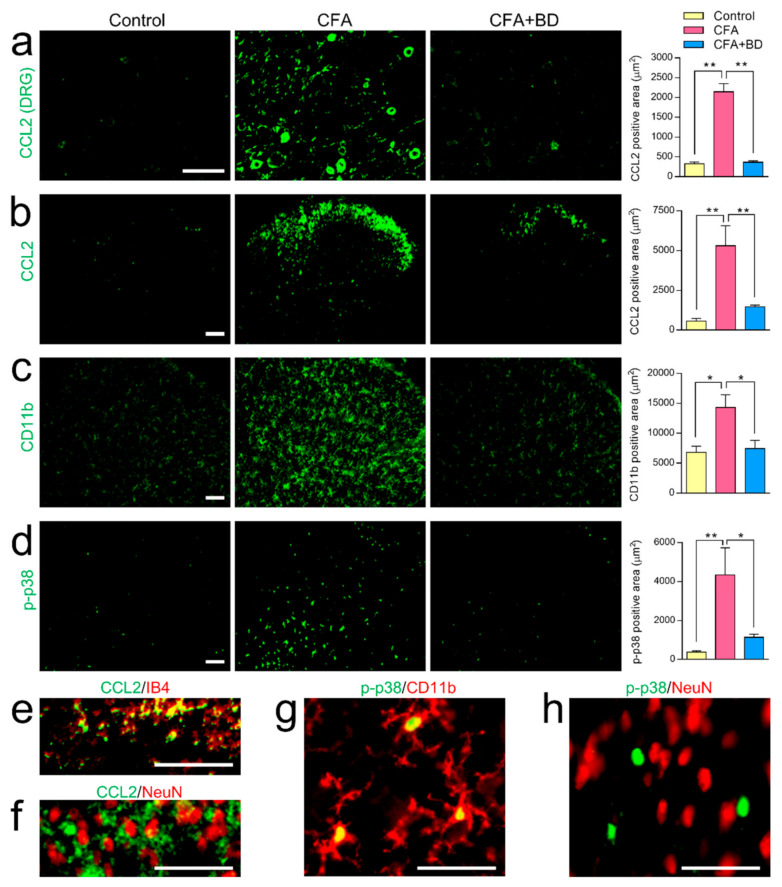
The effect of BD1047 (BD) on complete Freund’s adjuvant (CFA)-evoked CCL2 elevation and microglial activation. On the 7th day after CFA injection CCL2 expression was significantly higher in both DRG (**a**) and spinal dorsal horn (**b**) than in non-inflamed control group. CCL2 expression was dramatically reduced by repeated BD administration. CFA-induced inflammation produced a marked increase in the amount of CD11b (**c**) and phosphorylated p38 mitogen-activated protein kinases (p-p38, (**d**) present in the spinal dorsal horn. These increases were also dramatically inhibited by repeated treatment of BD. * *p* < 0.05, ** *p* < 0.01; Tukey’s multiple comparison test; three rats in each group; Scale bar = 100 µm. Double-labeling study showing that CFA-induced CCL2 immunoreactivity in lamina I-II spinal cord predominantly overlapped with IB4 (**e**) but not with NeuN (**f**). CFA-induced p-p38 immunoreactivity in the spinal dorsal horn was exclusively co-localized with microglia (CD11b, (**g**)) but not with neurons (NeuN, (**h**)). Scale bar = 50 µm.

Immunohistochemical observations made the 7th day after CFA-induced inflammation revealed significantly increased CCL2 expression in the ipsilateral side of lumbar 4–5 DRG compared with the non-inflamed control, and that this inflammation was dramatically inhibited by BD1047 (100 mg/kg) (Figure 4a). Increased expression of CCL2 was also observed in lamina I-II spinal dorsal horn following CFA-induced inflammation, which was alleviated by BD1047 treatment (Figure 4b). CFA also significantly increased p-p38 expression (Figure 4d) as well as expression of spinal CD11b (Figure 4c), but this was blunted by BD1047. Despite these observed effects, GFAP immunoreactivity was the same in the non-inflamed rats and the CFA-injected rats, suggesting that the CFA itself was not responsible for any increased activation of astrocytes (data not shown). CFA-induced CCL2 expression in the lamina I-II spinal dorsal horn was co-localized with unmyelinated PAFs marker IB4 (Figure 4e) but not with spinal neuronal marker NeuN (Figure 4f), indicating peripheral specific expression of CCL2. CFA-induced p-p38 expression also strongly overlapped with microglia marker CD11b (Figure 4g) but not with NeuN (Figure 4h), suggesting that CFA elicited p-p38 elevation only in microglia but not in neurons at the spinal cord level.

### 2.5. The Change of Sig-1R Immunoreactivity in DRG following CFA-Induced Inflammation

**Figure 5:**
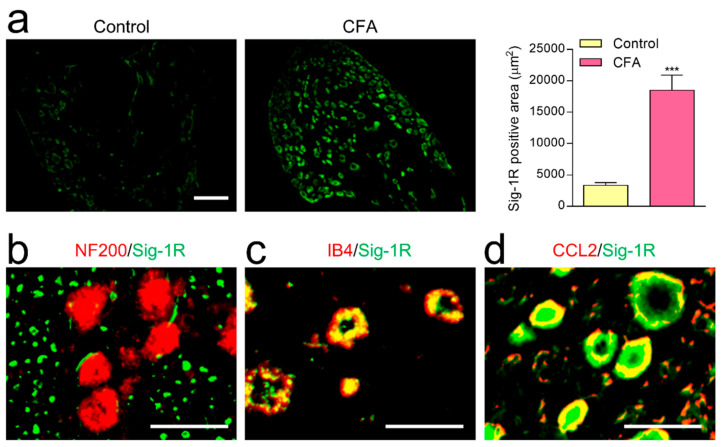
The distribution of sigma-1 receptor (Sig-1R) in DRG neurons after CFA-induced inflammation. (**a**) In DRG, Sig-1R immunoreactivity on the 7th day after CFA-induced inflammation was significantly higher than in the non-inflamed control rats (3 rats in each group; scale bar = 100 µm, *** *p* < 0.001; Tukey’s multiple comparison test). Double-labeling study showing Sig-1R immunoreactivity extensively overlapped with IB4 (**c**) and CCL2 (**d**), but not with NF200 (**b**). Scale bar = 10 µm.

We found that, by the 7th day after CFA-induced inflammation, Sig-1R immunoreactivity in DRG was significantly higher than in the non-inflamed control rats (Figure 5a). Sig-1R in DRG was expressed extensively within IB4 (Figure 5c) but not within NF200 as a myelinated PAFs marker (Figure 5b). Moreover, the majority of Sig-1R is colocalized with CCL2 (Figure 5d), suggesting that elevated Sig-1R plays a regulatory role in CCL2 release in unmyelinated PAFs.

## 3. Discussion

Intraplantar injection of zymosan, an immunogenic trigger, elicits thermal and mechanical hyperalgesia, a property that has invited its extensive use in research designed to evaluate anti-nociceptive effects and underlying mechanisms relevant to the treatment of rheumatoid arthritis [18,19]. As zymosan-induced hyperalgesia develops, the microglia activation and subsequent release of interleukin-1β in the spinal level are considered as important mediators [20,21,22]. In a recent study, we reported that CCL2′s immunoreactivity was selectively increased in IB4-positive unmyelinated PAFs but not in spinal neurons and glia cells following intraplantar zymosan injection in rats [13]. Present DRG culture study reveals that co-incubation of zymosan dose-dependently increases CCL2 release from IB4-positive DRG neurons and that this increase is dramatically reduced by BD1047. The results suggest that the peripheral influence of Sig-1R plays a substantial role in the release of zymosan-induced CCL2 from unmyelinated PAFs. In a subsequent in vivo experiment, we observed that the oral administration of BD1047 reduced zymosan-induced hyperalgesia, CCL2 over-expression in DRG neurons, and microglia activation (via CD11b and p-p38) in the spinal dorsal horn, suggesting that the anti-nociceptive effect of BD1047 may predominantly be attributed to its direct inhibition of CCL2 released from unmyelinated PAFs under zymosan-induced inflammatory conditions.

Indeed, both CCL2 from PAFs and its cognate receptor CCR2 in microglia are closely involved in pronociception [12], in that the intrathecal injection of chemokine produces hyperalgesia by binding to its microglia-located receptor [23]. Moreover, intrathecal administration of CCL2 in a naive animal produces hyperalgesia, which may be induced by neuronal sensitization of NMDA receptors resulting from microglial activation and cytokine release [24]. In our previous study, we demonstrated that zymosan-induced hyperalgesia was predominantly reduced by intrathecal pre-treatment with minocycline (an inhibitor of microglial activation), SB203580 (a p38 inhibitor), and interleukin-1 neutralizing antibody [7]. In that study, we also reported that the intrathecal injection of RS504393 (a CCR2 antagonist) was capable of reducing zymosan-induced hyperalgesia, as well as elevated levels of CD11b, p-p38, and interleukin-1β in the spinal dorsal horn [13]. Our subsequent electrophysiological study clarified that minocycline was predominantly responsible for inhibition of both the CCL2-evoked inward current in substantial gelatinosa neurons and the increased level of p-p38 in microglia [13]. These overall results strongly suggest BD1047′s peripheral effect of inhibiting CCL2 release alleviates zymosan-induced hyperalgesia through microglia-evoked neuronal activation.

Our previously disclosed experimental results have demonstrated that even a single treatment with BD1047 is capable of producing significant anti-nociceptive effects in acute pain models [3,4,5,7]. Based on these previous findings, in the present study, we evaluated whether repeated administration of BD1047 reduced CFA-induced hyperalgesia. We confirmed that repeated oral administration of BD1047 over the course of the 7 days post-CFA injection significantly reduced thermal hyperalgesia and mechanical hyperalgesia. CFA-induced inflammation upregulates CCL2 immunoreactivity in unmyelinated PAFs, but not in the components of the spinal cord [14]. In that respect, our results suggest that BD1047′s inhibitory effect on peripheral sensitization is the result of a decrease in CCL2 activity in unmyelinated PAFs following CFA-induced pain.

Similar to our zymosan model, repeated treatment with BD1047 significantly reduced CFA-induced microglia proliferation and p-p38 elevation in the spinal dorsal horn. We also observed that CFA-induced p-p38 expression was exclusively co-localized with microglia and not spinal neurons. Following the unilateral intra-articular injection of CFA in rats, repeated intrathecal administration of microglia inhibitor (minocycline) significantly suppresses the induction of hyperalgesia and activation of spinal microglial activation [25]. Intrathecal injection of CCL2 produces sustained hyperalgesia through microglia activation, indicating that both CCL2 from PAFs and CCR2 in microglia are closely involved in the formation of the pain sensation [12]. Indeed, it was shown that the p-p38 present in microglia facilitates its release of TNF-α and brain-derived neurotrophic factor, which in turn bind to nociceptive neurons to induce central sensitization regarding the chronic pain state [26]. In this context, our current findings suggest that BD1047 may reduce CCL2-induced microglia activation and subsequent nociceptive neuronal sensitization in CFA-induced pain models.

Sig-1R is highly expressed in different pain areas of the brain (i.e., amygdala and the periaqueductal grey), spinal cord and peripheral nervous system including DRG [27]. In our previous study, we used a single-cell RT-PCR to detect the presence of Sig-1R mRNA in DRG and spinal lamina I-II neurons after zymosan-induced inflammation [7]. On the basis of this finding, we propose that the nociceptive strength of Sig-1R may be increased in both DRG and spinal neurons under inflammatory conditions. In the present study, Sig-1R expression in DRG neurons, which is rarely found in non-inflamed rats, was significantly increased 7 days from the time of intra-articular CFA injection, suggesting that CFA-induced inflammation triggers upregulation of Sig-1R in DRG neurons. The present study also observes that CFA-induced inflammation dramatically increases Sig-1R immunoreactivity in CCL2-positive unmyelinated PAFs. Although the role of Sig-1R in spinal neurons, as well as brain areas following systemic administration of BD104, remains unclear, it does seem to play a role in regulating CFA-induced hyperalgesia by directly inhibiting CCL2 through the blockage of Sig-1R in IB4-positive unmyelinated PAFs. Accordingly, peripheral blockage of Sig-1R in unmyelinated PAFs may play a critical role in mediating the anti-nociceptive effect associated with the systemic administration of BD1047 and CFA-induced inflammation.

Peripheral inflammation, including that associated with rheumatoid arthritis, results in intractable pain and may cause various health issues in our life. Current treatments to control rheumatoid arthritis and chronic pain conditions are incapable of meeting these goals over sustained timeframes. Notably, it was reported on the basis of several phase I-II clinical studies that chronic administration of Sig-1R antagonists results in fast absorption, rapid distribution, and slow elimination (comparable to one day of administration) below an acceptable safety threshold [28,29]. CCL2 is a well-known effective stimulant of mechanosensitive unmyelinated PAFs in rat skin [30], while BD1047 is known as a direct blocker for Sig-1R agonist-mediated CCL2 elevation in human microglia [31]. Preclinical and clinical findings further suggest that the blockade of CCL2 is an important target in the design of potential treatments for rheumatoid arthritis-induced pain [14,20]. In this context, Sig-1R antagonists are promising analgesics, in that their inhibitory mechanism covers CCL2-mediated microglia–neuronal sensitization in the spinal cord.

**Figure 6:**
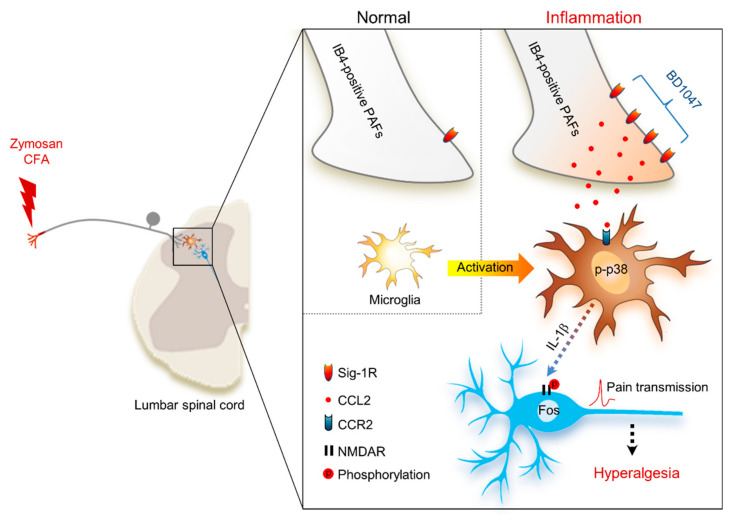
Schematic diagram proposing that the anti-nociceptive mechanism of sigma-1 receptor (Sig-1R) antagonist BD1047 for the inhibition of CCL2-induced microglia-neuron interactions in the spinal cord. Under inflammatory conditions, the presence of Sig-1R (a plausible binding site of BD1047) is especially increased in IB4-positive unmyelinated primary afferent fibers (PAFs). The activation of Sig-1R post-inflammation facilitates the elevation of CCL2 synthesis and release in unmyelinated PAFs. CCL2 binds to its plausible binding site (CCR2) in microglia, which elevates levels of phosphorylated p38 mitogen-activated protein kinase (p-p38) in microglia. CCL2-induced interleukin-1β (IL-1β) release from the microglia ultimately increases the spinal neuronal activation (NMDA receptor (NMDAR) phosphorylation and Fos expression) associated with hyperalgesia.

Figure 6 summarizes the interaction between BD1047 and CCL2 on the basis of our present and previous findings. In this schematic: (1) Peripheral inflammation increases Sig-1R population (i.e., CFA) and CCL2 release in unmyelinated PAFs; (2) BD1047 can peripherally inhibit CCL2 release in IB4-positive unmyelinated PAFs; (3) The inhibition of CCL2 release, in turn, reduces spinal microglia activity (via p-p38 elevation and interleukin-1β release); (4) Reduction of microglia activation ultimately reduces spinal neuronal activity (via phosphorylation of NMDA receptor NR1 subunit and Fos expression) associated with hyperalgesia.

## 4. Materials and Methods

### 4.1. Experimental Animals

Male Sprague-Dawley rats (Dae-Han Biolink Co., Eumsung, Korea) were housed in colony cages with free access to food and water and maintained in temperature and light controlled rooms (23 ± 2 °C, 12/12 h light/dark cycle with lights on at 08:00) for a minimum of 7 days prior to the commencement of any experiments. Three-week-old rats were used for the DRG culture study, while seven-week-old rats were used in the zymosan- and CFA-induced inflammation studies. All of our experiments were approved by the Animal Care and Use Committee at Jeonbuk National University (Approval number: JBNU 2016-47) and conformed to NIH guidelines (NIH publication No. 86-23, revised in 1985).

### 4.2. Primary Culture of Dorsal Root Ganglion with Zymosan

Rats were terminally anesthetized and the DRGs of both sides were dissected quickly from segments L1–L5. The ganglia were washed in 1 mL Nutrient Broth (NB) minimal medium and centrifuged at 250× *g* for 2 min. Medium was changed 3 times and 0.25% Trypsin-EDTA was added into 2 mL NB minimal medium in a 15 mL tube within a 37 °C water bath over 8 min. The 15 mL tube containing the DRG neurons and NB minimal medium was centrifuged at 250~500× *g* for 2 min, after which 1 mL of NB medium was added to the resulting pellet and clumps of cells were very gently broken. The cells were centrifuged again after titration, and the resuspended cell solution was filtered through a 45 µm cell strainer. The cells were stained with Trypan Blue and counted under a microscope. Isolated DRG cells (2 × 10^4^) were cultured in NB medium onto glass coverslips coated with poly-(L-lysine) (100 mg/mL) in a 24-well plate and kept for 2 days at 37 °C in a 5% CO**_2_** incubator. After 2 days, the NB medium was changed in the 24-well plate (500 µL/well), at which point various concentrations of zymosan (0, 0.1, 0.3, 1 and 3 µg/mL) and/or BD1047 (10 µM) were applied in the 24-well plate for 3 h. After reaction, 200 µL medium was centrifuged at 1000× *g* for 5 min at 4 °C, and supernatants were carefully transferred to a new tube for anti-rat CCL2 ELISA assay (R&D system, MN, USA). A cultured coverslip was fixed with 4% paraformaldehyde fixation for immunostaining. These experiments were performed in triplicate.

### 4.3. Induction of Peripheral Inflammation and Drug Administration

Rats were lightly anesthetized with 3% isoflurane in a mixed N_2_O/O_2_ gas. Inflammation was induced by either a single subcutaneous injection of zymosan (Sigma, MO, USA) (6 mg/200 µL in sterile saline) into the plantar surface of the right hind paw or a single intra-articular injection of CFA (50 µg *Microbaterium butyricum* (Difco, MI, USA)/50 µL in mineral oil) into the unilateral ankle articular cavity of the right hind paw. A 23-gauge needle was inserted from the gap between the tibiofibular and tarsus bone into the articular cavity.

BD1047 (Tocris, Bristol, UK) was dissolved in the physiological saline. Oral administration of BD1047 (10 mg/kg, 30 mg/kg and 100 mg/kg) was performed 1 h before the zymosan injection. In the CFA model, repeated oral treatment with BD1047 (10 mg/kg, 30 mg/kg and 100 mg/kg; one time per day) was performed between days 1–7 post-CFA injection. Physiological saline was administrated to the vehicle control group.

### 4.4. Pain Behavioral Assay

Animals were acclimatized to the testing chambers for 1 h the day before and the day of inflammation induction. Pain behavioral tests were performed at 0 h (before zymosan injection) to obtain a baseline and 1, 3 and 5 h after drug administration. In the CFA model, pain behavioral tests were performed daily at 0 day (before CFA injection) to obtain a baseline, as well as 3 h after treatment with drugs.

Thermal hyperalgesia: Using a thermal plantar tester (Ugo Basile, Varese, Italy), radiant heat was applied to the plantar surface of the hind paw until the rat lifted its paw. A photoelectric cell automatically turned the heat source off when the reflected light beam was interrupted (i.e., when the animal withdrew its paw). The time at which this occurred was recorded as the paw withdrawal latency. Heating was automatically terminated after 20 s to prevent tissue damage in the event an animal failed to withdraw its paw prior to the cutoff. The thermal threshold in a normal animal was 18–20 s. All values were measured in duplicate with a minimum interval of 3–5 min between two successive measurements.

Mechanical hyperalgesia: The nociceptive flexion reflex was quantified with an Analgesy meter (Ugo Basile), a device that generates a mechanical force that increases linearly over time. The force was applied to the dorsum of the rat’s hind paw by a wedge-shaped plunger. The nociceptive threshold is defined as the force (g) at which the rat withdraws its paw. The mechanical threshold in a normal animal was 180–200 g.

### 4.5. Western Blotting Assay

Three hours after the injection of zymosan, three rats from each of the control, zymosan-vehicle, and zymosan-BD1047 (100 mg/kg) groups had the right side of their L_4-5_ spinal dorsal horn (lamina I-V) or L_4-5_ DRG removed under anesthesia with 5% isoflurane. The spinal cord or DRG was homogenized in buffer containing 1 M Tris (pH 7.5), 1% NP-40, 0.5 M EDTA (pH 7.5), 50 mM EGTA, 1 M dithiothreitol, 1 M benzanidine and 0.1 M PMSF. The total amount of protein in each sample was determined using a Bradford dye assay before each was loaded onto polyacrylamide gels. The tissue homogenates (30 µg protein) were separated by 10% SDS-polyacrylamide gel electrophoresis and transferred to nitrocellulose. After washing the blots with TBST (10 mM Tris–HCl (pH 7.6), 150 mM NaCl, 0.05% Tween-20), the membranes were blocked with 5% skim milk for 1 h. The samples were incubated with primary antibodies for rabbit anti-CCL2 (Millipore, 1:1000), rabbit-Iba1 (Wako, VA, USA, 1:1000), and rabbit anti-p-p38 MAPK (Cell Signaling, 1:500). β-actin antibody was used as a loading control (Sigma). After the secondary antibody reaction, the bands were visualized with enhanced chemiluminescence (Amersham Pharmacia Bio-tech, Bukinghamshire, UK).

### 4.6. Immunohistochemistry and Image Analysis

Three hours after zymosan injection or on the 7th day after CFA injection, three rats from each of the control, zymosan (or CFA)-vehicle, and zymosan (or CFA)-BD1047 (100 mg/kg) groups were selected and deeply anesthetized with 5% isoflurane and perfused transcardially with calcium-free Tyrode’s solution followed by fixative containing 4% paraformaldehyde in 0.1M phosphate-buffered saline (PBS, pH 6.9). The L_4-5_ spinal cord or the right side of the L_4-5_ DRG was removed immediately after perfusion, post-fixed in the same fixative for 4 h, and then cryoprotected in PBS containing 30% sucrose (pH 7.4). A series of frozen sections (40 µm thickness in the spinal cord, 20 µm thickness in DRG) were cut using a cryostat.

Spinal cord sectioning was performed by free-floating protocol. DRG sections, sections from spinal cord slices, and DRG culture coverslips were generated by thaw mounting protocol. Preblocking with 5% appropriate normal serum according to the origin of the primary antibody was performed in 5% BSA and 0.3% Triton X-100 in PBS, after which the sections were incubated at 4 °C overnight in either rabbit anti-CCL2 (Millipore, MA, USA, 1:1000), mouse anti-CD11b (Serotec, Oxford, UK, 1:1000), rabbit anti-p-p38 MAPK (Cell Signaling, MA, USA, 1:500), biotin-conjugated anti-isolectin B4 (IB4, Sigma, 1:10,000), mouse anti-NF200 (Sigma, 1:10,000), mouse anti-GFAP (Milli-pore, 1:10,000), or rabbit anti-Sig-1R (Santa Cruz Biotechnology, CA, USA, 1:1000). The sections were subsequently incubated in Alexa Fluor 568-conjugated host-specific secondary antibody (Invitrogen, CA, USA, 1:200) for 2 h at room temperature. For immunofluorescence double staining, sections were first incubated with primary antibodies and visualized with Alexa Fluor 488 conjugated secondary antibodies (1:200, Invitrogen). Other primary antibodies were visualized with Alexa Fluor 568.

Slides were scanned with an ECLIPSE 80i (Nikon Corporation, Tokyo, Japan) fluorescent microscope, and three sections were selected from each animal. Images of individual sections were digitized with 4096 grayscale levels using a cooled CCD camera (CoolSnap ES, Photometrics, AZ, USA) connected to a computer-assisted image analysis system (MetaMorph, Universal Imaging Co., Downingtown, PA, USA). To increase spatial resolution, slices were imaged over a distance of 10 µm within an image-stack along the z-axis (z-stack, stacks distance) followed by three-dimensional deconvolution using the AutoQuant algorithm in Metamorph. To maintain a constant threshold for each image and compensate for subtle variability in the immunostaining, we calculated positive immunoreactivity only as a threshold area at least 200% brighter than the average grayscale level of each image.

### 4.7. Statistical Analysis

Data values were expressed as the mean ± SEM. All data were analyzed using the commercially available software GraphPad Prism 6 (Graphpad Software, San Diego, CA, USA). In the pain behavioral study, statistical analysis was carried out using Two-way analysis of variance (ANOVA) for repeated measures followed by Bonferroni’s Multiple Comparison Test (for Vehicle and BD1047) or Tukey’s multiple comparisons (for BD1047 dose–response analysis). Bonferroni’s Multiple Comparison Test (for CCL2 release in the DRG culture study,) or Tukey’s multiple comparisons (for image analysis in Western blotting and immunohistochemistry) were used.

## 5. Conclusions

We demonstrated that BD1047, by acting on unmyelinated PAFs, reduces inflammation-induced CCL2 release and subsequent microglia-dependent pain facilitation pathways. Our results suggest that the peripheral blockage of Sig-1R via CCL2 may be a promising approach to achieving an analgesic effect for the treatment of inflammatory pain diseases including rheumatoid arthritis.
